# Connecting the empirical dots on antipsychotic exposure and treatment in clinical high-risk for psychosis

**DOI:** 10.1017/S0033291725102225

**Published:** 2025-10-27

**Authors:** Andrea Raballo, Michele Poletti, Antonio Preti

**Affiliations:** 1 University of Lugano Faculty of Biomedical Sciences: Universita della Svizzera italiana, Lugano, Switzerland; 2 Department of Mental Health and Pathological Addiction, Child and Adolescent Mental Health Service, Reggio Emilia Local Agency – IRCCS Advanced Technologies and Care Models in Oncology, Reggio Emilia, Italy; 3 Department of Neuroscience, University of Turin: Universita degli Studi di Torino, Turin, Italy

The recent article by Mukhtar et al. (2025) reporting on predictors of antipsychotic (AP) inception in AP-naïve youth at clinical high-risk for psychosis (CHR-P) represents an important advance, being AP need between baseline enrolment and follow-up an almost neglected issue in CHR-P studies. By restricting analyses to AP-naïve CHR-P individuals at baseline and focusing on first AP initiation within 6 months, the study provides a cleaner lens on prescribing practices. Yet, the true significance of this methodological stance becomes clearer when situated against the accumulated evidence on pharmacological exposures in CHR-P and the trajectory of conceptual refinements over the past decade.

Mukhtar et al. (2025) treat AP inception as an endpoint of interest. Prior work has emphasized that new AP prescribing in CHR-P should be conceptualized as a *functional equivalent* of psychometric transition (Raballo, Poletti & Carpenter, 2019; Raballo, Poletti, & Preti, 2020), reflecting clinician-perceived worsening necessitating pharmacological containment. This reframing is critical because it clarifies that AP inception is not a neutral event but rather a clinically meaningful risk marker, similar in weight to transition itself.

North America Prodrome Longitudinal Study (NAPLS) sample was commendably restricted to AP-naïve youth. This is not an incidental design choice: over successive analyses, it has become increasingly incontrovertible that baseline AP exposure confounds prognosis and inflates transition risk estimates (Raballo et al., 2020). The more recent demonstration of a *dose–response effect*, whereby higher baseline AP doses predict proportionally greater transition risk (Raballo, Poletti, & Preti, 2024), sharpened this point. Most recently, a secondary analysis of the multinational PSYSCAN cohort (Tognin et al., 2025) has reinforced the case (Raballo, Poletti, & Preti, 2025a): even *transient pre-baseline AP exposure* (TPAE) (≤30 cumulative days at subtherapeutic doses) doubled transition risk compared with AP-naïve participants (28% vs. 12%). This operationalization of TPAE underscores that even minimal AP need at inception functions as a potent prognostic specifier. The NAPLS focus on AP-naïve participants therefore reflects not only good methodological practice but also the logical consolidation of a line of converging empirical signals.

Mukhtar et al. (2025) report predictors of APs start over 6 months. Yet, meta-analytic work demonstrated that the prognostic weight of AP exposure peaks at 24 months and extends up to 48 months (Raballo, Poletti & Preti, 2023a). Integrating this perspective would contextualize the NAPLS 6-month window as an early signal within a longer risk arc, reinforcing the call for time-sensitive, adaptive intervention strategies.

Moreover, Mukhtar et al. (2025) found major depression a key driver of AP initiation. This coheres with prior findings (Raballo, Poletti & Preti, 2023b), which showed baseline antidepressant exposure is associated with lower transition risk, unlike AP exposure. The contrast underscores a critical practice-guideline gap: while psychotherapeutic interventions and SSRIs remain first-line, APs are still commonly prescribed for depressive comorbidity despite limited preventive benefit. Moreover, the strong predictive effect of benzodiazepine use in NAPLS resonates with meta-analytic results linking their exposure to higher symptom severity and poorer outcomes (Livingston et al., 2025). In this framework, concomitant psychotropics like benzodiazepines must be explicitly documented, both as potential proxies of acuity and as prognostic modifiers (Raballo, Poletti, & Preti, 2023c). Systematic reviews have documented pervasive under-reporting of baseline pharmacological exposures in CHR-P research (Raballo, Poletti, & Preti, 2025b). The NAPLS paper makes progress but could more fully integrate the transparency checklist proposed in this literature to set a new reporting standard for large cohorts.

In conclusion, NAPLS offers valuable insights into prescribing patterns in AP-naïve CHR-P youth. Its focus on AP-naïve participants is best understood not as an isolated methodological refinement but as the cumulative consequence of a decade-long trajectory of empirical clarifications around the prognostic meaning of AP exposure. When read alongside dose–response and temporal dynamics analyses, the PSYSCAN-derived construct of transient pre-baseline AP exposure, and antidepressant versus AP prognostic profiles, the broader lesson emerges: AP inception is a functional marker of heightened clinical severity, and transparent handling of pharmacological exposures is indispensable for precise CHR-P modeling. Bridging these threads enriches the interpretation of NAPLS and guides next-generation preventive psychiatry studies.
